# Nursing students’ experience of learning cultural competence

**DOI:** 10.1371/journal.pone.0259802

**Published:** 2021-12-17

**Authors:** Isabel Antón-Solanas, Elena Tambo-Lizalde, Nadia Hamam-Alcober, Valérie Vanceulebroeck, Shana Dehaes, Indrani Kalkan, Nuran Kömürcü, Margarida Coelho, Teresa Coelho, Antonio Casa Nova, Raul Cordeiro, Lucía Sagarra-Romero, Ana B. Subirón-Valera, Isabel Huércanos-Esparza

**Affiliations:** 1 Department of Physiatry and Nursing, Faculty of Health Sciences, Universidad de Zaragoza, Zaragoza, Spain; 2 Instituto de Investigación Sanitaria, Hospital Universitario Miguel Servet, Zaragoza, Spain; 3 Faculty of Health Sciences, Universidad San Jorge, Zaragoza, Spain; 4 Servicio Aragonés de Salud, Miguel Servet Women’s and Children’s Hospital, Zaragoza, Spain; 5 Department of Nursing, AP University of Applied Sciences and Arts, Antwerpen, Belgium; 6 Faculty of Health Sciences, Istanbul Aydin University, Istanbul, Turkey; 7 School of Education and Social Science, Instituto Politécnico de Portalegre, Portalegre, Portugal; 8 School of Health Sciences, Instituto Politécnico de Portalegre, Portalegre, Portugal; 9 Faculty of Health Sciences, Villanueva de Gállego, Universidad San Jorge, Zaragoza, Spain; National Taiwan University of Science and Technology, TAIWAN

## Abstract

**Introduction:**

European societies are rapidly becoming multicultural. Cultural diversity presents new challenges and opportunities to communities that receive immigrants and migrants, and highlights the need for culturally safe healthcare. Universities share a responsibility to build a fair and equitable society by integrating cultural content in the nursing curricula. This paper aims to analyze European student nurses´ experience of learning cultural competence and of working with patients from diverse cultural backgrounds.

**Materials and methods:**

A phenomenological approach was selected through a qualitative research method. 7 semi-structured focus groups with 5–7 students took place at the participants’ respective universities in Spain, Belgium, Turkey and Portugal.

**Results:**

5 themes and 16 subthemes emerged from thematic analysis. Theme 1, concept of culture/cultural diversity, describes the participants’ concept of culture; ethnocentricity emerged as a frequent element in the students’ discourse. Theme 2, personal awareness, integrates the students’ self-perception of cultural competence and their learning needs. Theme 3, impact of culture, delves on the participants’ perceived impact of cultural on both nursing care and patient outcomes. Theme 4, learning cultural competence, integrates the participants’ learning experiences as part of their nursing curricula, as part of other academic learning opportunities and as part of extra-academic activities. Theme 5, learning cultural competence during practice placements, addresses some important issues including witnessing unequal care, racism, prejudice and conflict, communication and language barriers, tools and resources and positive attitudes and behaviors witnesses or displayed during clinical practice.

**Conclusion:**

The participants’ perceived level of cultural competence was variable. All the participants agreed that transcultural nursing content should be integrated in the nursing curricula, and suggested different strategies to improve their knowledge, skills and attitudes. It is important to listen to the students and take their opinion into account when designing cultural teaching and learning activities.

## Introduction

Currently, the world is experiencing the highest rate of human mobility ever recorded [1]. “Along with the recent trend of globalization and increasing cultural exchanges among countries”, multiculturalism is rapidly growing in European societies [2]. Cultural diversity enriches the societal aspect of European countries [1] and presents new challenges and opportunities to communities that receive immigrants and migrants [3]. Specifically within healthcare environments, the growing number of people moving to Europe emphasizes the need for safe care practices that address, and embrace, differences in worldviews, expectations, attitudes, communication styles and language [4]. Yet, patients from minority groups frequently experience worse quality care than the majority population [5]. In healthcare, a minority group is frequently associated with a specific, distinctive characteristic or attribute, generally nationality and/or ethnicity. However, many populations, “whether defined by race, ethnicity, immigrant status, disability, sex, gender” [6], geography, socioeconomic status and even age, experience worse quality care compared with the general population [6]. This may be due to their distinctive health characteristics and attributes, but also to different ways of understanding health and healthcare, different needs and different expectations of care and the healthcare service.

Healthcare professionals should be competent to care for patients, families and groups from different cultural backgrounds [7]. According to Shepherd [8], “when nurses provide culturally sensitive care, clients are more likely to report greater satisfaction with care”. Therefore, nurses need to be culturally mindful of their clients’ individual needs and adapt their practice in order to provide culturally safe and equitable care for all [9, 10]. However, nurses and other healthcare professionals may lack the knowledge, skills and attitudes necessary to provide equitable care for all, including those from a different cultural background [11]. Communication difficulties, in particular, pose significant difficulties in cross-cultural care encounters [12].

[title of project], is funded by the European Commission under Key Action 203 Strategic Partnerships for Higher Education [13]. It represents a collaboration between 4 European universities: [names of universities]. In this paper, we present the results from our investigation of European student nurses’ understanding and perception of cultural competence, their experience of learning in a multicultural context and that of working with patients from diverse cultural backgrounds.

### Background

According to Purnell and Paulanka [14], cultural competence is “developing an awareness of one’s own existence, sensations, thoughts, and environment without letting it have an undue influence on those from other backgrounds; demonstrating knowledge and understanding of the client’s culture; accepting and respecting cultural differences; and adapting care to be congruent with the client’s culture” [15]. Cultural competence is an essential component of nursing care. In this context, culturally competent nursing care integrates specific knowledge, skills and attitudes that guarantee appropriate and equitable care for all, including diverse patient populations [7, 16].

Providing culturally competent care has been associated with positive patient outcomes including improved nurse-patient communication [17], higher patient satisfaction [18] and better health status [19]. In addition, according to Cruz et al [20], cultural competence contributes to reducing health disparities in healthcare facilities and improving health equity [21]. In contrast, culturally indifferent care may lead to a misinterpretation of patients’ needs, inaccurate diagnoses and treatment errors [22, 23], and has been linked to increased health disparities [24].

In previous years, the nursing community has focused on cultural competence of both students and qualified nurses [20]. According to the International Council of Nurses (ICN) [25], patients have the right to culturally and clinically appropriate care in order to ensure best patient outcomes. However, preparing nurses to provide safe and culturally competent nursing care requires significant education and training [26, 27]. Thus, the means of providing safe and culturally competent quality care should be central components of nursing education [7, 28]. Yet, previous studies have suggested that student nurses often lack the confidence to provide culturally competent care [29, 30]. Furthermore, the concept of transcultural nursing care continues to be insufficiently addressed in the field of nursing education [1, 31].

As future qualified nurses practicing in an ever growing and changing multicultural society, European student nurses must be equipped with essential knowledge, skills, values and attitudes that allow them to provide culturally mindful, safe and equitable care for all. Yet, little is known about how student nurses learn and experience cultural differences (and similarities) both at college and in practice [26, 32]. It is important to understand these experiences and perceptions in order to adapt nursing curricula to the student nurses’ learning needs. Thus, this investigation aims to:

- Identify the student nurses’ perceived level of cultural competence and establish whether transcultural nursing content is currently being integrated in undergraduate nursing programs.
- Describe the student nurses’ experience of learning cultural competence.
- Analyze the student nurses’ experience of working with patients from diverse cultural backgrounds.

## Materials and methods

### Design

A phenomenological approach was selected in order to illicit the student nurses’ perceptions and experience of learning cultural competence. This is appropriate as this methodological approach allows researchers to understand complex phenomena through the participants’ perspectives and meaning that they give to their lived experiences [33, 34]. We used the COREQ reporting guidelines in both the framing and presentation of methods and findings of this study [34].

### Participants

We recruited a total sample of 40 undergraduate student nurses registered in a Bachelor of Nursing program from one of the following universities [university 1] in Spain (12 students), [university 2] in Belgium (11 students), [university 3] in Turkey (10 students), and [university 4] in Portugal (7 students). We used a purposive sampling technique ensuring a good balance across the following characteristics: age, gender, nationality and year of study. Inclusion criteria for taking part in the study included:

- Undergraduate student nurses registered in one of the participating Higher Education Institutions (HEI).
- Student nurses who agreed to the conditions of the study and gave informed consent to participate.

### Data collection

Focus groups were selected as the most suitable method of data collection as they allow researchers to gain a rich understanding of participants’ perceptions, experiences, attitudes and beliefs [35]. In order to address the possibility of a power difference emerging during the group discussions due to the participants seniority level, the focus groups conductors ensured that a significant level of participation was obtained from each and every participant. 7 semi-structured focus groups with 5–7 students were conducted by an academic working at each of the study sites in the participants’ first language. All the student nurses from each university shared a language, namely Spanish or Portuguese or Dutch or Turkish. Similarly, the researchers that conducted the interviews were also proficient or native speakers or Spanish or Portuguese or Dutch or Turkish. Data collection took place between March and August 2019 and had an average duration of 30–60 minutes. The sessions were audio-recorded and transcribed verbatim. The original transcriptions were subsequently translated into English by the academic conducting the focus group, all of whom were proficient English speakers. All the researchers used the same (previously agreed) focus-group guide developed by I.A-S., L.S-R. and I.H-E. Focus-group questions addressed topics such as the influence of cultural difference on health, the students’ perceived level of cultural competence, their experience of learning about cultural issues in the classroom and their experience of learning in a multicultural environment. The questions in the interview guide were designed based a thorough review of the literature and our own personal experience of teaching and learning cultural competence [36] (Table 1).

**Table 1:** Topic guide for the focus-groups.

Opening question
We are interested in hearing about your experience of working with patients from diverse cultural backgrounds. Please can you tell us about any experiences you have had to date?
Follow up questions
Do you think cultural difference affects health (i.e. ethnicity, nationality, religion, etc.)? How? Do you think the needs of patients belonging to minority groups are being met currently in health care services? What impact do you think being from a diverse cultural background has on patients/families/groups? Have you ever considered how your own culture influences your nursing practice? Are you confident in your level of cultural competence to look after patients from diverse cultural backgrounds? How do you deal with difficulties/conflicts emerging from working with patients from different cultural backgrounds? Have you had any formal training and/or experiential learning in cultural competence? Are cultural issues addressed during lectures? How and when are these issues addressed? Does the university offer any other alternatives to improve your level of cultural competence? Do you think nursing lecturers are sufficiently prepared to deliver teaching in this area? What do you think are your needs in the way of education/training in cultural competence? Can you tell us about your experience of learning in a multicultural environment (if any)?

The following variables were collected in order to describe the sociodemographic and cultural characteristics of the sample: age (years), gender, marital status, occupation, race/ethnicity, religious affiliation, adherence to religion, residential environment, socioeconomic level, country of study, year of study, language competence, cultural competence training, involvement with diverse patients/organizations, experience working with patients from diverse backgrounds, and experience living/studying abroad for at least 3 months. A copy of this questionnaire is provided as (S1 Table).

### Data analysis

Descriptive statistics was used to analyze the sociodemographic and cultural characteristics of the sample; qualitative variables were analyzed using frequency and percentage whilst quantitative variables were analyzed using mean and standard deviation. Qualitative data were analyzed using the NVivo software (Version 12, QRS International).

Two researchers, E.T-L. and I.H-E, analyzed the anonymized transcripts separately following Braun and Clark’s [37] phases for thematic analysis: 1) familiarizing with data, 2) generating initial codes, 3) searching for themes, 4) reviewing themes, 5) defining and naming themes and 6) producing the report. In addition, the following techniques were used in order to guarantee quality:

- Nurse students from four European HEIs were recruited in order to contrast and compare the findings across multiple sites.
- During the process of data collection, the researchers involved took field notes including personal reflections on the participants’ response and on specific points arising from the focus groups.
- During the process of data analysis, an audit trail of the researchers’ both independent and collaborative decision-making process was kept.
- During the process of writing the final report, frequent contact between the authors from the 4 study sites was maintained in order to oversee the process of data analysis and identify potential biases.

5 Themes and 16 subthemes were derived from the data. A selection of some of the most illustrative and representative verbatims from the focus groups is presented as in S2 Table. We made a conscious effort to offer a balanced picture of the students’ testimonies by ensuring that the opinions of the students from every study site were represented both in the text and S2 Table. Thus, the codes assigned to each participant do identify their university of origin. However, any references to specific places, health services etc., were removed from the text in order to safeguard the participants’ personal identity. However, the choice of verbatims was also guided by their content and meaning and, for this reason, it is possible that not all countries are represented in all the themes and subthemes. It would have been interesting to elaborate a comparative analysis of our findings by university of origin, but the results were interpreted in an integrated manner.

### Ethical considerations

Approval from [name of REC] (REC) was sought and obtained (study reference number 16–2019). This resolution was translated into English and sent, along with the original resolution, the study protocol, the participant information leaflet and the consent form, to all the participating HEIs. All the HEIs involved in this investigation evaluated these documents separately and each university gave explicit permission to collect data. Specifically, permission was granted to implement this study by the local RECs at [name of university] ([website]) and [name of university] ([website]). At [name of university] the process was slightly different and followed the flowchart in the document enclosed. All three institutions accepted the resolution from the Spanish REC and, therefore, no further local REC approvals were issued nor required. Consent form was given by the participants, who were adults, in writing.

## Results

The sociodemographic and cultural characteristics of our sample are presented in Table 2. The average age of the student nurses was 22.5 years. Most of the participants were female (72.5%). Regarding their marital status, 70% were single and 30% were either married or in a stable relationship. The vast majority of the student nurses defined themselves as having a white ethnic background (97.51%), being middle social class (95%) and were studying full time (82.5%). All of the participants were studying in their birth country at the time of being interviewed. Almost 83% of our participants had not had any prior training in cultural competence, and the same percentage of student nurses stated that they did not belong to a culturally diverse family or group of friends. Finally, only a minority had lived or studied abroad for over three months (12.5%).

**Table 2:** Sociodemographic and cultural characteristics of the sample (n = 40).

Participants’ characteristics	N (%) or Mean (SD)
**Age (years)**	40 (22.5)
**Languages spoken other than mother tongue**	40 (1.46)
**Sex**	
Male	11 (27.5)
Female	29 (72.5)
**Marital status**	
Single	28 (70)
Married/partner	12 (30)
**Occupation**	
Study only	33 (82.5)
Work and study	7 (17.5)
**Race/Ethnicity**	
Caucasian/White	39 (97.5)
Other	1 (2.5)
**Religious affiliation**	
Catholicism	17 (42.5)
Islam	11 (27.5)
Atheism/None	12 (30)
**Adherence to religion**	
Practicing	18 (45)
Non-practicing	22 (55)
**Residential environment**	
Urban	27 (66.6)
Rural	13 (33.3)
**Socioeconomic level**	
High social class	1 (2.5)
Middle social class	38 (95)
Low social class	1 (2.5)
**Country of birth**	
Belgium	11 (27.5)
Portugal	7 (17.5)
Spain	12 (30)
Turkey	10 (25)
**Country of study**	
Belgium	11 (27.5)
Portugal	7 (17.5)
Spain	12 (30)
Turkey	10 (25)
**Year of study**	
First year	1 (2.5)
Second year	4 (10)
Third year	17 (42.5)
Fourth year	18 (45)
**Belonging to a culturally diverse family or group of friends**	
Yes	7 (17.5)
No	33 (82.5)
**Prior cultural competence training**	
Yes	7 (17.5)
No	33 (82.5)
**Prior or current voluntary work with people from diverse cultural background**	
Yes	5 (12.5)
No	35 (87.5)
**Experience in caring for patients from diverse cultural background**	
Yes	29 (72.5)
No	11 (27.5)
**Lived/studied abroad for at least 3 months**	
Yes	5 (12.5)
No	35 (87.5)

### Theme 1. Concept of culture/cultural diversity

This theme integrates the students’ perceptions of the role of culture in society, as well as the implications of living in a multicultural society.

#### Meaning of culture

A variety of descriptions and definitions of culture were identified. Some of our participants believed that culture was associated with aspects such as nationality, race, and ethnicity, whilst others made reference to other elements such as lifestyle, religion and tradition.

*‘Ethnic group’*

*(IPP*-*07)*

*‘After all*, *culturally*, *we very often start thinking about distinguishing between color and religion*, *or indeed religion*, *but from the cultural point of view*, *there can already be a Belgian and a German*, *there is a huge difference in culture between them’*

*(APU*-*02)*

*‘Tradition*, *beliefs and norms’*

*(IAU*-*06)*

#### Identifying cultural diversity

Frequently, the concepts of multiculturality and cultural diversity were used interchangeably; cultural diversity in particular was associated mainly with nationality and a specific way of acting or behaving in society.

*‘Mix of different types of cultures*, *different types of behaviors and seeing what happens when you mix them all*, *that combination’*

*(USJ*-*11)*

*‘Our society is very colorful—we had individuals from different cultures and background in our country even before the Syrian migrants came in’*

*(IAU*-*05)*

#### Ethnocentricity

The participants often referred to their own cultural norms and habits as the “right” way to act and behave, which could sometimes result in “cultural conflict” between people from different cultural backgrounds. Further, they suggested that belonging to a different culture represents a challenge when it comes to understanding the needs of the other.

*‘We are always me*, *me*, *me*; *we think that our culture is the best for everyone [everyone nods]*. *Then*, *you assume that*, *unless you see a physical or religious difference*, *you feel that everyone does the same things as you*, *then you assume that everyone is going to do things the way you do things’*

*(USJ*-*02)*

*‘The different cultures are always clashing with each other’*

*(USJ*-*12)*

### Theme 2: Personal awareness

This theme includes the participants perception of their own level of cultural competence and their identified learning needs.

#### Self-perception of cultural competence

This subtheme integrates the students’ perception of their own cultural competence based mainly on their experiences of looking after patients and their relatives during clinical placement. Whereas some of them did feel comfortable with their perceived level of cultural competence, most of them considered that they still had much to learn. Some of the students made emphasis on their lack of knowledge about, and understanding of, specific minority cultures, whereas others suggested that a lack of cultural knowledge could be overcome by showing an empathic, respectful and open attitude towards those who were different, and by confronting these situations with a predisposition to learn and adapt one’s practice.

*‘Yes*, *I am reassured in my own level of cultural competence*, *for me now*, *especially after several years of internship experience in a multicultural context*. *I can manage my care’*

*(APU*-*09)*

*‘¡Ay*! *I don’t know if it’s adequate [the student’s level of cultural competency]*, *but when I care for a person from a different culture*, *I base my actions on respect and*, *if I don’t know*, *I just ask’*

*(USJ*-*12)*

#### Perception of learning needs

The students identified a lack of practical experience and/or exposure to culturally diverse patients, and gaps in their knowledge of issues relating to cultural competence, as areas of improvement in relation to the perceived need to provide culturally sensitive and safe nursing care.

*‘I’d like to have more training on this [cultural competence]*. *[*…*] To have some more information in case they come to clinic and you know nothing about their culture’*

*(USJ*-*11)*

*‘I need a way to communicate with patients and behave more professionally in case of cultural conflicts or issues related to it’*

*(IAU*-*04)*

### Theme 3: Impact of culture…

This theme comprises the students’ perception of the impact of culture on the care delivered by both qualified and student nurses to patients who belong to the same or a different culture, and also the impact of different cultural customs and habits on people’s’ health.

#### …On caring

The participants felt that cultural difference affected care decisions; they described how their own cultural background, and that of their mentors, had an impact on their practice and thus affected patient care. Cultural difference in this context was sometimes described as ethnic difference; on the other hand, culture and religion were cited as factors which contribute to improve the quality of nursing care.

*‘The first thing that comes to mind is definitely mine and yes*, *I think it [the student’s own culture] influences everything*; *the way I speak or the way I approach a patient or ask him questions’*

*(USJ*-*12)*

*‘As per our religion and culture*, *cleanliness and hygiene are very basic requirements and important*. *I think this perspective influences my nursing practice in a positive way’*

*(IAU*-*01)*

#### …On health

The participants agreed with the notion that culture affected health and disease, and described cultural practices and behaviors which had both a positive and a negative impact on people’s health. It was often the case that the practices and customs deriving from the students’ own culture were perceived as being generally beneficial to health:

*‘Different lifestyles are determined by different cultures and can improve or worsen your health status*, *for example*, *the health of the Spanish society in general is good due to the Mediterranean diet’*

*(USJ*-*09)*

However, examples were also given of specific cultural behaviors which were different from their own and which were perceived as being positive:

*‘On a level of social interaction*, *in terms of the visit of your family or a large presence of family or involvement*. *[*…*] I think that the involvement of that family and those closer social connections is certainly an advantage compared to that more individual culture we have here’*

*(APU*-*02)*

Religion was frequently cited by as heavily influencing patients’ decisions mostly in a negative way, and the student nurses provided a myriad of different examples:

*‘The health staff warned her*, *but she was very adamant and did not have the abortion due to her religious belief and went home’*

*(USJ*-*05)*

Other factors perceived by the students as exerting a negative influence on patients’ choices and, thus, health include language and traditional customs:

*‘Some cultural practices may have adverse effects on health*. *I witnessed a Syrian patient who made her new*-*born baby sleep in a swaddle prepared by her*. *The swaddle was so stiffly tied that it could adversely affect the baby’s hip development or displacement of the hip joint—such cultural practices could be harmful to the new*-*born’*

*(IAU*-*03)*

### Theme 4: Learning cultural competence

The student nurses described their experiences of learning cultural competence either as part of their bachelor studies, as part of extracurricular learning opportunities provided by their respective HEIs or as part of activities taking place outside the university.

#### As part of the nursing curriculum

This subtheme integrates the participants’ descriptions of the nursing courses which, in their opinion, addressed cultural competence. Only one fourth year, optional course, delivered at [University 1] was described by the student nurses as focusing primarily on cultural competence; instead, most of the participants confirmed that cultural competence was addressed occasionally as part of a range of different modules.

*‘You have those—nursing on the move—(online modules)*, *but I found that very limited’*

*(APU*-*02)*

*‘For example*, *it was discussed in Ethics a bit’*

*(USJ*-*11)*

*‘For example*, *the teachers of Socio*-*anthropology*, *Ethics and Psychology of Health deal with these issues*

*(IPP*-*06)*

*‘Not as a separate course but as a part of other course as anthropology or interpersonal communication’*

*(IAU*-*06)*

The participants identified mainly two types of teaching and learning activities used when addressing cultural competence in the classroom, namely clinical case-studies and clinical simulation. In most cases, the students found these teaching and learning activities insufficient.

*‘There were news*, *we saw the news and all that but*, *you know*…, *it was all very theoretical’*

*(USJ*-*08)*

*‘We do case studies and conduct open discussion on problems relating to intercultural issues in class’*

*(IAU*-*02)*

The students appraised their teachers’ expertise to teach about cultural competence. Most of the students thought that their teachers’ knowledge was adequate to integrate this content in their respective courses, or to find the information necessary to do so. Interestingly, they did not see the need for all their teachers to acquire the necessary knowledge and skills (and attitudes) to teach cultural competence. Instead, they suggested that cultural content should only be integrated in those modules with a more “social” orientation.

*‘Some instructors are sufficiently prepared and deliver lectures in this area*, *though not all’*

*(IAU*-*01)*

*‘Some subjects do not require this (*…*)*. *In Technical subjects*, *such as biology*, *that is not really necessary’*

*(APU*-*04)*

Finally, the participants mentioned strategies and alternatives to improve their level of cultural competence. Specifically, they mentioned clinical simulation with real patients from diverse cultural backgrounds, role-play techniques, courses in communication and (cultural) conflict resolution and increasing the presence of cultural competence in the nursing curricula.

*‘I would offer a course about communication skills’*

*(USJ*-*01)*

*‘I would like to learn about situations*, *case studies about such situations but I would also like to be able to experience them “in reality”*. *To have contact with these cases*. *To talk to the people*, *to learn real stories*. *Or we could also do study visits’*

*(IPP*-*05)*

#### As part of other academic activities

The participants recognized extracurricular learning opportunities offered by their respective HEIs about cultural competence. However, most of them considered these learning opportunities insufficient, or said that they were not disseminated widely enough. Having said this, some did acknowledge a lack of personal initiative on their part either to actively seek or participate in said activities.

*‘You have those—nursing on the move—(online modules)*, *but I found that very limited*. *Because it really was a lesson and you were done’*

*(APU*-*02)*.

*‘There are many things*, *like the Erasmus program too*, *like that*, *you can spend two weeks in England*, *and there an International Department and you can get information there if you show initiative’*

*(USJ*-*10)*

In addition, the students shared their experiences as exchange students, mainly through the Erasmus+ program, and as participants in summer schools and other complementary courses and/or cultural immersion activities. All the student nurses who had taken part in these experiences described them as enriching and an opportunity to open one’s mind. This is despite their initial fears and concerns about different issues, including their personal capacity to manage themselves in a multicultural environment.

*‘I participated in a transcultural summer school in [name of location] in 2018*. *I came across students and teachers from some parts of Europe as well as met refugees from Pakistan and Afghanistan [*…*]*. *This cultural interaction was very fruitful and a great learning experience for me’*

*(IAU*-*03)*

Interestingly, some of the students suggested that cultural competence could also be learnt from their own culturally diverse peers, simply by being in a multicultural environment.

*‘While discussing the adaptation process and difficulties faced by our foreign classmates’*

*(IAU*-*07)*

#### As part of activities taking place outside the university

The students also described learning opportunities external to their respective HEIs including voluntary work, experiencing cultural encounters as part of their professional and/or social activities and even playing videogames.

*‘I volunteered to give yoga lessons to children who were different*, *I mean*, *there were Spanish children too*, *but there Muslims*, *Gypsies and so on’*

*(USJ*-*01)*

### Theme 5: Learning cultural competence during practice placements

Clinical placements provided cultural encounters and learning opportunities that the students recognized and generally valued. This theme integrates the students’ perception and experience of caring for culturally diverse patients, including the perceived barriers or difficulties to provide culturally mindful and safe nursing care [10], and the strategies employed to overcome them.

#### Shortfalls and differences in the care provided to culturally diverse patients

The participants observed and described scenarios in which the quality of nursing care was suboptimal, resulting in care which only partially addressed the needs of culturally diverse patients. The nurses’ workload, the language barrier, a lack of specific resources (or knowledge of these resources) and the certain degree of prejudice against a specific group of people, for example Muslims in Belgium and Gypsy Roma in Spain, were sometimes cited as barriers to provide safe and mindful nursing care that met the patients’ needs and expectations.

*‘Because now there is no male nurse available to care for that man*, *but he has to get care*, *and maybe he needs to receive care for his intimate parts as well*, *from wound care or something like that and then that won’t happen and that can get infected’*

*(APU*-*09)*

*‘The health service needs of the Syrian refugee community are being met adequately*, *however*, *the other minority communities may not have been receiving the same amount of health care’*

*(IAU*-*01)*

The Belgian students made reference to specific regulations and barriers at healthcare system level which had a significant impact on patients’ access to care:

*‘In [name of hospital] that sometimes happens*. *People who actually need surgery*, *but they are not allowed to have surgery because they don’t have any papers in Belgium*. *So*, *we have to send them back home’*

*(APU*-*09)*

Finally, it is worth describing some of the Turkish student nurses’ view of health inequity in Turkey, where the Syrian refugees are seen as having better access to care than other minorities and even the majority Turkish population.

*‘The healthcare needs of the Syrian refugee community are being met adequately*. *However*, *the other minority communities may not have been receiving the same amount of health care’*

*(IAU*-*01)*

*‘Syrian inpatients are being hospitalized as and when necessary*, *the Turkish patients are often being sent home with the excuse that there are not enough beds’*

*(IAU*-*03)*

#### Racism and prejudice in the healthcare service

The student nurses witnessed racist, prejudiced and discriminatory attitudes towards not only patients and their relatives, but also towards healthcare professionals whilst on placement. These negative attitudes were observed in the students themselves, the staff and also the patients.

*‘An African nurse who does something wrong in the eyes of a Belgian old lady*, *who is then charged with malpractice more quickly than if I had made that mistake*. *Me being Belgian—with the right colour*, *so to speak’*

*(APU*-*09)*

*‘I have seen loads of racism against patients [*…*]*; *nasty comments behind their back*. *Sometimes I even*…, *I don´t confront them*, *but sometimes I have made a cutting remark*, *like saying*: *“don’t cross the line’*

*(USJ*-*02)*

*‘And then there are the “positive” comments like*: *´look how nice they are even though they are Gypsies’*. *It’s like*…, *what*?!*’*

*(USJ*-*02)*

#### Communication and language barriers

This subtheme comprises the student nurses’ perceptions and experiences of communication barriers during practice placement. Generally, the language barrier was perceived as a key challenge to communicate with diverse patients.

*‘The language barrier is the single most important thing in the picture during clinical placements’*

*(APU*-*02)*

*‘Communication is the basic problem*. *The health care staff cannot serve the patient properly or understand his health requirement due to language barrier’*

*(IAU*-*06)*

However, language was not the only communication barrier observed or experienced by the students. Sometimes, the nurses’ attitude when caring for people from a different cultural background was cited as a source of difficulty too:

*‘I have also experienced some miscommunication myself*, *mostly a lack of communication between the nurse and other cultures sometimes*. *They don’t seem to make the effort*, *or they don’t dare say something*, *and this results in miscommunication later on’*

*(APU*-*04)*

The students reflected on the impact of these barriers on the quality of patient care and the nurse-patient relationship.

*‘I witnessed a case with a foreign inpatient for gastric reduction surgery where nursing care was incomplete because of lack of communication*. *The patient was in a depressive state but could not express herself*. *Also*, *psychological counselling could not be given because of the language barrier’*

*(IAU*-*02)*

*‘They may not be able to trust the health care staff due to language barrier*. *They may be suspicious of the health care received*

*(IAU*-*01)*

In addition, they perceived that it was necessary to make an extra effort to breach this rather frequent and significant language barrier. However, there was a certain level of disagreement about who had to make that extra effort:

*‘The language barrier is an issue that must be overcome even from the patient’s side*. *In other words*, *they should make an effort to learn the language of the country that they have come to live in´*

*(IAU*-*07)*

*‘Because of the language barrier the foreign patients are often scared to ask questions*. *As health care staff we must provide them with adequate information about the treatment even if they don’t ask for it’*

*(IAU*-*10)*

#### Cultural conflict

The students experienced conflictive or difficult situations during the practice placements emerging between the healthcare staff and the patients from a different cultural background. Conflict was often associated with aspects such as eating habits, religion and gender issues.

*‘I have also had friction in connection with eating habits’*

*(IAU*-*04)*

*‘She got very angry because it happened a few times already*. *But I think that maybe rules should be made about prayer*-*times’*

*(APU*-*06)*

*‘He didn’t want to be treated by any women*, *just men*. *For example*, *we went to insert his urinary catheter and he didn’t let us’*

*(USJ*-*03)*

One of the Spanish students described how, on one occasion, conflict was resolved through the intervention of a cultural mediator; however, this was not the rule but the exception in their discourse:

*‘I think that cultural mediators are super important and people just don’t know of them*. *Even I didn’t know they existed until one day that for me it was like*: *“wow*!*” If I ever find myself in a similar situation*…, *cultural or whatever*, *I will call a mediator because*, *you know*, *this is like seeing the light*; *a solution’*

*(USJ*-*01)*

#### Tools and resources

The students described a range of tools and resources, used and witnessed during clinical placements, intended to overcome the (mainly) linguistic communication barrier. Some of these tools and resources were more adequate than others. For example, the students described working with professional interpreters:

*‘Here there are always interpreters in the house*, *in the hospital*. *Especially in [name of hospital]*, *interpreters come to translate’*

*(IAU*-*09)*

Yet, they were not always available:

*‘But I think that’s where the language barriers come in*, *if people don’t understand you well and you don’t find any interpreters*, *then good luck trying to explain it*! *[*…*] Sometimes I find that very frustrating’*

*(APU*-*05)*

In these cases, they often resorted to other individuals to interpret for the patients including cleaning and other staff:

*‘Sometimes people from the cleaning crew translate for us as they often are from another origin as well*. *So that’s also more convenient for us’*

*(APU*-*09)*

The students themselves were sometimes asked to interpret for a patient using a language other than their mother tongue, usually English:

*‘They came to me this morning*. *We have a Russian patient who doesn’t speak Spanish and none of the nurses spoke English and they come to me and say*: *“come here and see if you can manage because we just can’t understand each other”‘*

*(USJ*-*04)*

More worrying still is the fact that the patients themselves, and their relatives, were asked to interpret for other patients when professional interpreters were not available:

*‘Often there are other patients who speak the same language*. *We utilize their help also from time to time’*

*(IAU*-*01)*

*‘The relatives also can help’*

*(USJ*-*07)*

Frequent reference was made by the student nurses to a range of different technologies used to communicate with patients who did not speak the same language:

*‘We had a patient who didn’t speak English but there’s something interesting and good about the new technologies*, *and the translators online*, *and the internet*. *He’d write in his mobile phone what he wanted to say and translate it into Portuguese*, *in the translator*, *and we‘d do the same with him*. *It was a way of communicating with him’*

*(IPP*-*04)*

However, the results were not always satisfactory:

*‘He didn’t speak Spanish*, *nor English*; *we didn’t speak Russian*, *so we used Google translate*. *We typed the words in Google and clicked and the phone spoke in Russian and all that*, *but it was awful*. *You can’t establish a good nurse*-*patient relationship*, *you know*? *It was completely different*, *you felt like*…, *you know*? *Like*…, *insecure’*

*(USJ*-*03)*

Other tools and resources used included pictograms, gestures and other non-verbal communication strategies and even music:

*‘Well*, *we had this linguistic barrier*. *All the communication was made with mimics*, *gestures*, *like “nooo”*, *“that’s OK” (thumbs up) and things like that’*

*(IPP*-*04)*

*‘I saw once in ICU that they had various scales with pictures so that all the patients had to do was point to one picture or another*, *but they told me that they never used them*; *they were there for decoration’*

*(USJ*-*09)*

*‘In the intensive care children unit*, *out of the 28 patients*, *26 were Syrian refugee children*; *the nurses had put on Arabic music to entertain the children’*

*(IAU*-*07)*

#### Positive attitudes and behaviors

The students described certain attitudes and behaviors which were described as positive and conducive to a better nurse-patient relationship, as well as higher quality nursing care; in short, a more equitable nursing care for all:

*‘There is nothing more unequal than treating everyone the same’*

*(USJ*-*01)*

Specific values, skills and attitudes were mentioned, including active listening, respect, trust, empathy, focusing on the patient’s needs and avoiding prejudice.

*‘I really do ask my patients about it*. *So*: *“what is this like for you*, *in your culture”*? *With Moroccan families*, *for example*, *or Islamic culture*, *how do they feel about taking care of the body of someone who is deceased*, *and other things*? *[*…*] All I do is ask*, *so that I know more about what to do*, *the next time I find myself in such a situation*? *That helps me a lot’*

*(APU*-*09)*

*‘Don’t treat patients as you would like to be treated but as they would like to be treated’*

*(USJ*-*05)*

## Discussion

This article analyzed the perceptions and experiences of cultural care of a sample of Belgian, Spanish, Portuguese and Turkish undergraduate student nurses.

All of our participants had been born in the same country where they were studying and the vast majority defined themselves as being from a white ethnic background, middle social class and studying full-time. This implies that our sample was largely representative of the majority population. This seems also to be the case in previous qualitative studies on the same topic [2, 38]. With regard to the students’ previous exposure to culturally diverse people and experience of caring for patients from different cultural backgrounds, it was similar to that reported in previous similar recent research [7, 38].

The participants expressed different opinions and definitions of the concept of culture and cultural diversity; whereas some student nurses associated culture primarily with ethnicity and nationality, others were able to offer a wider description of the concept encompassing aspects such as lifestyle, religion and tradition. This wider definition of culture has also been described in previous studies [15, 39, 40] and was adopted throughout this investigation. Interestingly, a certain tendency to ethnocentrism became apparent in the student nurses’ discourse, who sometimes identified their own culture with the “norm” and the normal way of acting and behaving. According to Amiri and Heydari [41], it is not infrequent for both qualified and student nurses to behave in an ethnocentric way towards patients from different cultures. Unfortunately, ethnocentric attitudes and approaches in health care may adversely affect patient care and lead to worse patient outcomes [42]. Thus, undergraduate nursing programs should address these issues in order to limit the possibility of student nurses adopting ethnocentric attitudes and behaviors not only towards their culturally diverse patients but also their colleagues.

The students’ self-perception of cultural competence ranged from adequate to inadequate, with some participants suggesting specific strategies and attitudes to overcome their perceived lack of competence including respect, empathy and active listening. Previous studies have analyzed student nurses’ self-perception of cultural competency, describing it as poor to moderate [29, 43], with the students perceiving that their ability to provide culturally congruent care gradually increased throughout their training, leading to implications of the need for continued education relating to this concept [43]. Our participants were able to express specific learning needs and gaps in their training regarding their ability to provide culturally and linguistically appropriate care, including a lack of practical experience and exposure to culturally diverse patients, both in a controlled setting such as clinical simulation and on placement. Perhaps more importantly, they analyzed the impact of culture not only on their own practice, but also on their patients’ health, and made specific recommendations on methods for integrating cultural competence in the nursing curricula. This is important as, as suggested by Sumpter and Carthon [44], giving the students the opportunity to express and discuss their opinions on this matter through focus groups may help to identify specific, student-centered teaching and learning methods to integrate this content in the curriculum.

Our participants confirmed that the integration of cultural content in their respective nursing curricula was not consistent and mostly insufficient. Generally, the students reported what can be described as a “casual” approach to cultural issues in the classroom, with teachers addressing the topic occasionally during lessons, frequently through examples and case-studies. Use of case-studies to illustrate cultural issues in healthcare programs has been documented in the literature [45, 46]; other alternative teaching and learning strategies include international experiences [47], liaison with and participation of service users [48] and class-debate and discussion [44, 49], among others. However, teacher-led classroom activities were not the only learning opportunities described by our participants, who acknowledged extracurricular and even extra-academic activities leading to improved cultural knowledge, skills and attitudes. The Turkish and Belgian students especially suggested that specific knowledge, skills and attitudes relating to cultural competence could also be learnt from their own culturally diverse classmates, simply by being in a multicultural environment. Based on our experience of teaching and learning in a multicultural environment [10], as well as the students’ opinions, we recommend that cultural encounters are provided and promoted both as part of theoretical and clinical training of undergraduate nursing students [50], allowing time for debriefing in order to guide and encourage the students’ critical thinking and reflection [5].

The student nurses’ description of their experiences of caring for culturally diverse patients whilst on placement provided interesting insights into the way the students learn in practice. They recognized good practice, as well as what can be described as suboptimal nursing care, and suggested specific barriers hindering the provision of mindful and safe nursing care. These included the nurses’ workload, communication and language barriers, gender issues, lack of resources including professional interpreters and prejudice against specific groups such as Muslims in Belgium, Gypsy Roma in Spain and Syrian refugees in Turkey. These are arguably some of the most prevalent and visible barriers to equitable nursing care [51, 52]. From an educational perspective, it is important to realise that student nurses witness unequitable care whilst on placement more or less frequently. According to Djkowich et al [53], bearing witness in nursing practice can be contextualised as a moral and a political obligation. When student nurses bear witness of suboptimal or unequitable care, they must be supported to critically examine their understanding of key issues, such as power and prejudice. In fact, these authors go one step further and suggest that nurses who bear witness must also accept the “concomitant responsibility to take action to challenge injustice once we have borne witness to it” [53]. However, it must be taken into account that student nurses often find it difficult to challenge negative attitudes during practice placement. We suggest that both nursing faculty and mentors work collaboratively to identify challenges in the clinical learning environment and prepare the students to address them [54]. Further, healthcare training programs are responsible for and socially accountable to contribute to meeting the needs of each and every individual in a society. This implies recognising health inequities and designing and implementing measures to reduce disparities, including those related to cultural difference.

Whereas it was extremely interesting to analyze the perceptions and experiences of student nurses who belong to the cultural majority within their respective societies, we acknowledge that out study did not hear the voices of student nurses belonging to minority groups. It would be interesting to include participants from minority cultural backgrounds in future studies addressing these issues. Also, although we analyzed the student nurses’ testimonies as a whole, we acknowledge that specific differences exist in the way the students perceive, experience and learn about cultural competence both in the classroom and in practice. These differences emerge from the educational and healthcare systems, and the cultures and societies, represented in each country and must be taken into account when planning educational activities.

## Conclusions

Our findings suggest that the student nurses’ perceived level of cultural competence was variable. Yet, they were able to articulate their learning needs and suggest strategies to integrate transcultural nursing content in the undergraduate nursing curriculum. We recommend that the students’ voices are heard before implementing educational strategies to address cultural issues in the classroom. Also, we suggest that opportunities for discussion and critical reflection on these issues, particularly those emerging from practice, are provided to student nurses. Healthcare training programs should address health inequities and design and implement measures to reduce disparities, including those related to cultural difference.

## Supporting information

S1 Table Sociodemographic characteristics and cultural background of the participants. (DOCX)

S2 Table Selection of verbatims from the focus groups. (DOCX)
