# Supplementary material for: Nursing students’ experience of learning cultural competence
Source: PLoS One. 2021 Dec 17;16(12):e0259802. doi: 10.1371/journal.pone.0259802 (PMC8683022; doi:10.1371/journal.pone.0259802)
Supplement: S1 Table — (DOCX) [file pone.0259802.s001.docx]

**S1 Table. Sociodemographic characteristics and cultural background of the participants.**

| Sociodemographic characteristics | |
| --- | --- |
| Age (years) |  |
| Gender | Male |
|  | Female |
|  | Other (specify) |
| Marital status | Single |
|  | Married/partner |
|  | Divorced |
|  | Widower |
| Occupation | Study only |
|  | Work and study |
| Race/ethnicity | White |
|  | Black |
|  | Asian |
|  | Chinese |
|  | Mixed |
|  | Other (specify) |
| Religious affiliation | Catholicism |
|  | Protestantism |
|  | Islam |
|  | Judaism |
|  | Buddhism |
|  | Atheism |
|  | Other (specify) |
|  | None |
| Adherence to religion | Practicing |
|  | Non-practicing |
| Residential environment | Urban |
|  | Rural |
| Socioeconomic level | High social class |
|  | Middle social class |
|  | Low social class |
| Father’s level of education | Primary education |
|  | Secondary education |
|  | Vocational training |
|  | Bachelor degree |
|  | Master degree |
|  | Doctorate |
| Mother’s level of education | Primary education |
|  | Secondary education |
|  | Vocational training |
|  | Bachelor degree |
|  | Master degree |
|  | Doctorate |
| Country of birth | Belgium |
|  | Portugal |
|  | Spain |
|  | Turkey |
|  | Other (specify) |
| Country of study | Belgium |
|  | Portugal |
|  | Spain |
|  | Turkey |
| Year of study | First year |
|  | Second year |
|  | Third year |
|  | Fourth year |
| Clinical work experience | Yes |
|  | No |
| Clinical work experience (years) |  |
| Cultural background | |
| Mother tongue | French |
|  | Dutch |
|  | Portuguese |
|  | Spanish |
|  | Turkish |
|  | Other (specify) |
| Other languages (specify language and level of competence according to the Common European Framework of Reference for Languages: A1, A2, B1, B2, C1, C2) |  |
| Belonging to a culturally diverse family | Yes |
|  | No |
| Prior cultural competence training | Yes |
|  | No |
| Prior/current voluntary work with patients from diverse cultural backgrounds and organisations (i.e.NGOs, etc.) | Yes |
|  | No |
| Experience in caring for patients from diverse cultural backgrounds | Yes |
|  | No |
| Lived/studied abroad for at least 3 months | Yes (specify where and how long) |
|  | No |
