# Supplementary material for: Nursing students’ experience of learning cultural competence
Source: PLoS One. 2021 Dec 17;16(12):e0259802. doi: 10.1371/journal.pone.0259802 (PMC8683022; doi:10.1371/journal.pone.0259802)
Supplement: S2 Table — (DOCX) [file pone.0259802.s002.docx]

**S2 Table. Selection of verbatims from the focus groups.**

| Theme | Sub-themes | Selected quotes |
| --- | --- | --- |
| Concept of culture / cultural diversity | Meaning of culture | “Comes from your family, my habits and my own personal life, that it still has an influence on how I do conduct my life” (APU-04)  “After all, culturally, we very often start thinking about distinguishing between colour and religion, or indeed religion, but from the cultural point of view, there can already be a Belgian and a German, there is a huge difference in culture between them” (APU-02)  “Tradition, beliefs and norms” (IAU-06)  “Ethnic group” (IPP-07) |
|  | Identifying cultural diversity | “Diversity of existing cultures and how they relate to each other“ (USJ-STD-09)  “Mix of different types of cultures, different types of behaviours and seeing what happens when you mix them all, that combination” (USJ-11)  “Our society is very colourful – we had individuals from different cultures and background in our country even before the Syrian migrants came in” (IAU-05)  “After all, culturally, we very often start thinking about distinguishing between colour and religion, or indeed religion, but from the cultural point of view, there can already be a Belgian and a German, there is a huge difference in culture between them. And I notice that in the hospital, for example, German patients are very different in the way they deal with each other and in the way they approach their health” (APU-02) |
|  | Ethnocentricity | “Certain standard and everything that is not according to that standard, is wrong” (APU-02)  “We are always me, me, me; we think that our culture is the best of everyone [everyone nods]. Then…, you assume that, unless you see a physical or religious difference, you feel that everyone does the same things as you, then you assume that everyone is going to do things the way you do things” (USJ-02)  “But you could just not be paying attention to his [the patient’s] need and you don’t realize that you are doing something wrong, you know?” (USJ-10)  “But in a way, I feel that many people have a certain standard and everything that is not according to that standard, is wrong. And I think that that consciousness-switch is that actually things that are not standard are an enrichment. When you see the world with that point of view, you can only learn. But indeed, it starts with our own culture, and any culture is very often learned from that ‘standard ‘as if this standard-box is the most important and it is only right-one” (APU-02) |
| Personal awareness | Self-perception of cultural competence | “Yes (I am reassured in my own level of cultural competence), for me now, especially after several years of internship experience in a multicultural context. I can manage my care…” (APU-09)  “I think that will also depend on which culture you encounter, because all cultures you will never get to know. You're going to be able to focus on different cultures, which I'm going to understand a little better than others, but all of them? That is a lot. That's what I think”. (APU-08)  “Ay! I don’t know if it’s adequate [his level of cultural competency], but when I care for a person from a different culture, I base my actions of respect and, if I don’t know, I just ask” (USJ-12)  “I feel inadequate in this field”. (IAU-10) |
|  | Perception of learning needs | “We have to face these problems anyway, and we don't have any baggage/knowledge about that. I think that's a shame. […] It is a cultural aspect in itself and I think that there is still a shortage in our education about how people deal with dying” (APU-02)  “About how to react if I encounter this situation in real life“ (APU-09)  “I’d like to have more training on this [cultural competence]. […] To have some more information in case they come to clinic and you know nothing about their culture (USJ-11)  “I think it is still inadequate, not all issues are addressed. […] I need a way to communicate with patients and behave more professionally in case of cultural conflicts or issues related to it” (IAU-04)  “I feel inadequate in this field. I need training in developing my level of cultural competency. […] Further training in cultural competence will be helpful, possible inclusion of such a course in the curriculum will be perfect” (IAU-10) |
| Impact of culture | … On caring | “I have been told from childhood that 'everyone is the same for you, all equal, you give everyone a chance without prejudice and if something does happen, you'll see what happens…. And with that attitude, I'm going through life, also as a nurse“ (APU-05)  “African nurses work at a somewhat slower pace because it has also come from their culture of 'we do everything slowly” (APU-09)  “Yes, I often work in [name of hospital], and there are many different cultures, all the time. Also nurses from different backgrounds. You can see there is also a different way of working, because one ‘culture, so to speak,’ goes a lot faster than the other” (APU-09)  “The first thing that comes to mind is definitely mine and yes, I think it [his own culture] influences everything, the way I speak or the way I approach a patient or ask him questions” (USJ-12)  “As per our religion and culture, cleanliness and hygiene are very basic requirements and important. I think this perspective influence my nursing practice in a positive way” (IAU-01) |
|  | … On health | “Different lifestyles are determined by different cultures and can improve or worsen your health status, for example, the health of the Spanish society in general is good due to the Mediterranean diet” (USJ-09)  “On a level of social interacting, in terms of the visit of your family, or a large presence of family or involvement …. I think that it can be a stimulating factor for your health. You see that often with patients who are alone, for example, they will heal less quickly, less well healed because a person is not just a body, you are a person with your body, you have your mind, you have your social circle and that all works together on your healing. And I think that the involvement of that family and those closer social connections, is certainly an advantage compared to that more individual culture we have here” (APU-02)  “When they need to give consent […] and you don´t really know if he [the patient] has understood what may happen to him. Or maybe things like…, religion, no? Some religions forbid blood transfusions” (USJ-04)  “With a new language, a different culture, a different way of life, that must cause loads of stress and, in some cases, could impact on the way you live, and, in the long term, that may affect your health” (USJ-12)  “Some cultural practices may have adverse effects on health. I witnessed a Syrian patient who made her new-born baby sleep in a swaddle prepared by her. The swaddle was so stiffly tied that it could adversely affect the baby’s hip development or displacement of the hip joint – such cultural practices could be harmful to the new-born” (IAU-03) |
| 1. Learning cultural competence | 1. As part of the nursing curriculum | 1. “You have those - nursing on the move - (online modules), but I found that very limited” (APU-02) 2. “For example, it was discussed in Ethics a bit” (USJ-11) 3. “For example, the teachers of Socio-anthropology, Ethics and Psychology of Health deal with these issues” (IPP-06) 4. “Not as a separate course but as a part of other course as anthropology or interpersonal communication” (IAU-06) 5. “There are certain cases discussed and there you learn but not everything. Just really the basics, I think” (APU-10) 6. “There were news, we saw the news and all that but, you know…, it was all very theoretical” (USJ-08) 7. “Things that come up as you are talking about other things” (USJ-04) 8. “We do case studies and conduct open discussion on problems relating to intercultural issues in class” (IAU-02) 9. “Some instructors are sufficiently prepared and deliver lectures in this area, though not all” (IAU-01) 10. “Teachers who are more involved in this [cultural issues] and they try to integrate it into their modules, as something personal, and others that are either not aware or they don’t give it any importance” (USJ-02) 11. “Some subjects do not require this (…). In Technical subjects, such as biology, that is not really necessary” (APU-04) 12. “In “playing case” exercises, you could integrate more cases from cultural themes in it. Also, from someone who doesn’t speak the language” (APU-05) 13. “Actually, they should organize lessons about this, but then mandatory, not an exam or something, but just mandatory so that people go there, and then actually learn more about the cultures in nursing in general” (APU-03). 14. “I would offer a course about communication skills” (USJ-01) 15. “I would like to learn about situations, case studies about such situations but I would also like to be able to experience them “in reality”. To have contact with these cases. To talk to the people, to learn real stories. Or we could also do study visits” (IPP-05) |
|  | As part of other academic activities | “People from different cultures came to the university” (USJ-01)  “I had a bit of a chance because last year I indicated that I wanted to do an internship at the social services, because I wanted to see a completely different part of the hospital. And then I went to a homeless shelter in the [name of district]. And then you literally get everything. And that has been really an eye opener. In fact, I remained involved as a volunteer for a long time. And that has indeed been a very big experience for me” (APU-02)  “Yes, and the fact that we can go in Erasmus exchanges to other countries, although that is always optional, can give us some experience. And there are Erasmus students at school, and we have classes together, which allows us to contact other cultures and gives us some experience in terms of the interaction with people from other cultures” (IPP-07)  “I participated in a transcultural summer school in [name of location] in 2018. I came across students and teachers from some parts of Europe as well as met refugees from Pakistan and Afghanistan […]. This cultural interaction was very fruitful and a great learning experience for me” (IAU-03)  “On cultural day, countries from Africa, Syria, Iran had their music concerts” (IAU-07)  “We have speaking clubs in school where international students from all countries participate and exchange their culture and language with others” (IAU-08)  “International student days are held where food, music, attire and culture from all countries are exhibited by the foreign students. The students are provided with funds from the “Student Sports and Culture” department of our university to exhibit their cultural activities” (IAU-03)  “I suppose these things go unnoticed, I mean, I don’t get the information, like last year with the summer school that I didn’t get the information and I didn’t know I could participate. It’s like it goes unnoticed, like nobody gives it any value” (USJ-09)  “Okay, I think this has to do with your own interests in the sense that, if I’m interested in learning languages and communicate with people, then I see it, but this is me; it’s my own interest, you know? I think that if we are here is because we are interested, but it is not something that is integrated into a module, for example. No one tells you that you have to become interested in that [cultural issues] and take it into account, that’s missing” (USJ-10)  “There are many things, like the Erasmus program too, like that, you can spend two weeks in England, and there an International Department an you can get information there if you show initiative” (USJ-10)   1. “You could actually learn it through the students themselves” (APU-07) 2. “While discussing the adaptation process and difficulties faced by our foreign classmates” (IAU-07) |
|  | As part of activities taking place outside the university | “I volunteered to give yoga lessons to children who were different, I mean, there were Spanish children too, but there Muslims, Gypsies and so on” (USJ-01)  “None apart from our classroom, where we have classmates from Turkish republics like Azerbaijan, Uzbekistan, Turkmenistan, Tatarstan. We also have a few classmates from Iran and African countries. The other multicultural exposure we get is in hospitals working mostly with Syrian refugees and other minority groups” (IAU-06)  “Working in an assembly line. There were just Africans and Arabs […]. On my first year they were all men, and they didn’t talk to me and I…, well, we worked from 6 am to 3 pm and I was bored stiff. Then I tried to talk to them and…, at the beginning they were always reluctant, or made some comments like… [leaves the sentence unfinished]” (USJ-04)  “Yes because, okay, I, for example, like to play on my computer [everyone laughs] and there you meet people. I have played with Russians, with Latin-Americans…, even with Chinese! Then I don’t know, it’s like that interaction generates…, generates interest, no?” (USJ-05) |
| Learning cultural competence during practice placements | Shortfalls and differences in the care provided to culturally diverse patients | “I felt the health staff were a bit too rough on her in their attitudes [towards a Syrian refugee]” (IAU-06)  “Because now there is no male nurse available to care for that man, but he has to get care, and maybe he needs to receive care for his intimate parts as well, from wound care or something like that and then that won’t happen and that can get infected” (APU-09)  “There was a problem there; she could have contacted with the legal tutor or a relative or someone, but she didn’t. Then, you know, she had her hands full” (USJ-01)  “With regard to placement, I have noticed that when we have to make an effort to communicate with someone, we just…, we pass” (USJ-05)  “I also think that they are more often poorly portrayed [Muslims] than, for example, a Christian” (APU-07)  “I sometimes…, I told them once that I knew that that figure existed [intercultural mediators]” (USJ-04)  “In [name of hospital] that sometimes happens. People who actually need surgery, but they are not allowed to have surgery because they don’t have any papers in Belgium. So, we have to send them back home” (APU-09)  “The healthcare needs of the Syrian refugee community are being met adequately. However, the other minority communities may not have been receiving the same amount of health care” (IAU-01)  “Syrian inpatients are being hospitalized as and when necessary, the Turkish patients are often being sent home with the excuse that there are not enough beds” (IAU-03) |
|  | Racism and prejudice in the healthcare service | “Friends of mine that they have had to lunch in the linen room because they can't sit with the rest because they are only interns” (APU-07)  “An African nurse who does something wrong in the eyes of a Belgian old lady, who is then charged with malpractice more quickly than if I had made that mistake. Me being Belgian - with the right colour, so to speak” (APU-09)  “They [healthcare staff] were gossiping about a Moroccan lady, and they were saying that all Moroccans fake their symptoms, and they don’t feel as poorly as they want you to believe […]. It was weird because that lady, for example, didn’t speak Spanish; she was with her son and they looked down on her because…, because she was Moroccan, you know saying that she was feigning her symptoms and she wasn’t in so much pain” (USJ-09)  “But you can't be racist as a nurse, can you?” (APU-06)  “On the other hand, I also came to see a patient who said: 'Oh finally a European nurse by my bedside', and then someone from a different culture was just outside the door, and he heard that too. Then I really felt like, we're all nurses, we all do our jobs, what makes the difference?” (APU-02)  “I have seen loads of racism against patients […]; nasty comments behind their back. Sometimes I even…, I don´t confront them, but sometimes I have made a cutting remark, like saying: “don’t cross the line” (USJ-02)  “I have seen racists behaviours at the [maternity] hospital. A mother came with her baby who was black and they [the nurses] always handed me the little black babies to vaccinate…, not just because I like babies but also because… If a white baby came, then they [the nurses] took care of him, but if the baby was black then it was my job” (USJ-03)  “For example, I was holding a [foreign] baby once and a lady, a worker, said: why did you have to come to Spain?” (USJ-03)  “And then there are the positive comments like: ´look how nice they are even though they are Gypsies’. It’s like…, what?!” (USJ-02)  “There are differences between…, right now at the maternity there are two children, a Muslim and a…, I don´t know, from Zaragoza. They both need palliative care. Well, the local mum was treated with affection, so much better than the Muslim mum […]. I wasn´t comfortable with that” (USJ-02) |
|  | Communication and language barriers | “The language barrier is the single most important thing in the picture during clinical placements” (APU-02)  “Communication is the basic problem. The health care staff cannot serve the patient properly or understand his health requirement due to language barrier” (IAU-06)  “I have also experienced some miscommunication myself, mostly a lack of communication between the nurse and other cultures sometimes. They don’t seem to make the effort, or they don’t dare say something, and this results in miscommunication later on” (APU-04)  “A lot of them just don’t really know why they are being treated or haven’t received an explanation” (IPP-01)  “I witnessed a case with a foreign inpatient  for gastric reduction surgery where nursing care was incomplete because of lack of communication. The patient was in a depressive state but could not express herself. Also, psychological counselling could not be given because of the language barrier” (IAU-02)  “They may not be able to trust the health care staff due to language barrier. They may be suspicious of the health care received (IAU-01)  “The language barrier is an issue that must be overcome even from the patient’s side. In other words, they should make an effort to learn the language of the country that they have come to live in´ (IAU-07)  “Because of the language barrier the foreign patients are often scared to ask questions. As health care staff we must provide them with adequate information about the treatment even if they don’t ask for it” (IAU-10) |
|  | Cultural conflict | “I have also had friction in connection with eating habits” (IAU-04)  “She got very angry because it happened a few times already. But I think that maybe rules should be made about prayer-times” (APU-06)  “I am a man. There was this young gypsy girl and when I had to do the hygiene procedures, she wouldn’t let me” (IPP-04)  “He didn’t want to be treated by any women, just men. For example, we went to insert his urinary catheter and he didn’t let us” (USJ-03)  “I think that cultural mediators are super important, and people just don’t know of them. Even I didn’t know they existed until one day that for me it was like: ‘wow!’ If I ever find myself in a similar situation…, cultural or whatever, I will call a mediator because, you know, this is like seeing the light; a solution” (USJ-01)  “If you are more open to their culture, they will be more open to your culture” (APU-09)  “But I think that both parties have to come up with an honest answer from yes, that bothers me about you and that bothers me about you and that you can then come to a compromise together” (APU-09)  “I will come down to his level and speak to him calmly and slowly so that he is not threatened” (IAU-07)  “Well, I think that in such a situation the first step would be to understand the reason why the person is acting in the way she is acting. Why is the person being aggressive with us and insisting on a certain behaviour? We should try to understand the religion or the culture that lead the person to have those daily behaviours… maybe that can explain why, when we assess that patient, she is confused or agitated…" (IPP-03) |
|  | Tools and resources | “Here there are always interpreters in the house, in the hospital. Especially in [name of hospital], interpreters come to translate” (IAU-09)  “But I think that’s where the language barriers come in, if people don’t understand you well and you don’t find any interpreters, then good luck trying to explain it! […] Sometimes I find that very frustrating” (APU-05)  “Sometimes people from the cleaning crew translate for us as they often are from another origin as well. So that’s also more convenient for us” (APU-09)  “Yes, we used to have a Chinese worker and she once had to come up to the ward because…, there was a Chinese boy and nobody spoke Chinese and, of course, they came looking for…, and the problem was that not all the Chinese speak the same language because China is so big. Poor girl…, in the end they managed to understand each other!” (USJ-07)  “They came to me this morning. We have a Russian patient who doesn’t speak Spanish and none of the nurses spoke English and they come to me and say: ‘come here and see if you can manage because we just can’t understand each other’“ (USJ-04)  “Often there are other patients who speak the same language. We utilize their help also from time to time” (IAU-01)  “The relatives also can help” (USJ-07)  “We had a patient who didn’t speak English but there’s something interesting and good about the new technologies, and the translators online, and the internet. He’d write in his mobile phone what he wanted to say and translate it into Portuguese, in the translator, and we’d do the same with him. It was a way of communicating with him” (IPP-04)  “With the help of the internet translate program I eased the communication with the rest of the healthcare team. If not for me, she would have missed out on an important issue such as the vaccination program for the neonate” (IAU-03)  “He didn’t speak Spanish, nor English; we didn’t speak Russian, so we used Google translate. We typed the words in Google and clicked and the phone spoke in Russian and all that, but it was awful. You can’t establish a good nurse-patient relationship, you know? It was completely different, you felt like…, you know? Like…, insecure” (USJ-03)  “Well, we had this linguistic barrier. All the communication was made with mimics, gestures, like ‘nooo’, ‘that’s OK’ [thumbs up] and things like that” (IPP-04)  “I saw once in ICU that they had various scales with pictures so that all the patients had to do was point to one picture or another, but they told me that they never used them; they were there for decoration” (USJ-09)  “In the intensive care children unit, out of the 28 patients, 26 were Syrian refugee children; the nurses had put on Arabic music to entertain the children” (IAU-07) |
|  | Positive attitudes and behaviours | “There is nothing more unequal than treating everyone the same” (USJ-01)  “I really do ask my patients about it. So: ‘what is this like for you, in your culture?’ With Moroccan families, for example, or Islamic culture, how do they feel about taking care of the body of someone who is deceased, and other things? […] All I do is ask, so that I know more about what to do, the next time I find myself in such a situation? That helps me a lot” (APU-09)  “I would like to add that for me it is very important to be that step ahead concerning these cultural issues, because one day we can receive foreigners in our country and another day we can be the foreigners who are received in another country” (IPP-04)  “Don’t treat patients as you would like to be treated but as they would like to be treated” (USJ-05)  “I know that I have those ideas about the person or group, but I put them on hold till I get to know the person” (IPP-05)  “I think that we, as we are nurses, we learn how to simplify the language so that every patient can understand us, you know” (IPP-02)  “I gave him my trust, talked to him, like: “tell me about your life” (laughs). Then I told him: ‘what do you think, should we go ahead and give you that shot?’. And then he said: ‘okay, but you have to do it’. Then I said: ‘aha!’. I opened the way and…, it took me a while, you know? I spent nearly half an hour talking to him!” (USJ-01)  “I will try to help personally if I can […]. I will try to give moral support to the patient if necessary” (IAU-07) |
